# A rapid and efficient *in planta* assay for assessment of RNA editing factor performance

**DOI:** 10.1111/tpj.71154

**Published:** 2026-10-12

**Authors:** Elena Lesch, Dennis Brandt, Markus Schwarzländer, Till Ischebeck

**Affiliations:** ^1^ Green Biotechnology Department, IBBP—Institute for Biology and Biotechnology of Plants University of Münster Schlossplatz 8 48143 Münster Germany; ^2^ Plant Energy Biology Department, IBBP—Institute for Biology and Biotechnology of Plants University of Münster Schlossplatz 8 48143 Münster Germany

**Keywords:** *Nicotiana benthamiana*, mitochondria, plastids, RNA editing, pentatricopeptide repeat (PPR) proteins, RNA‐binding proteins

## Abstract

Land plant mitochondria and plastids are endosymbiotic organelles that possess their own, reduced genomes. Gene expression is largely regulated at the transcript level, predominantly by nuclear‐encoded proteins. Notably, up to thousands of cytidines in organellar RNAs are specifically edited to uridines, mostly restoring conserved genetic information. Key players in this process are nuclear‐encoded pentatricopeptide repeat (PPR) proteins, which bind target RNAs in a one‐PPR:one‐nucleotide manner, following a two‐amino‐acid‐based code. Cytidine deamination is catalyzed by intrinsic or trans*‐*acting DYW domains. The modularity and programmability of PPR‐RNA interactions make PPR editing factors attractive candidates for the development of biotechnological tools. However, RNA target selection is not determined by the ‘PPR code’ alone, hampering the generation of highly effective and specific editors. Refining such tools requires efficient assays to dissect the determinants of editor‐RNA interaction. Here, we introduce a rapid assay to assess PPR editor specificity through transient expression in the cytosol of *Nicotiana benthamiana*, which offers a large and diverse transcriptome that enables extensive off‐target editing. Profiling of these off‐targets provides comprehensive information on an editor's target selectivity. Compared with a previous setup in the model moss *Physcomitrium patens*, which relies on stable transformation, the required experimental time is reduced from months to days. We demonstrate the power of the system by characterizing the off‐targets of the moss editor PpPPR56. The new assay allows rapid functional assessment of editing factor variants and removes a bottleneck in understanding editor‐RNA interactions, which is a prerequisite for the rational improvement of PPR‐based RNA editing tools.

## Significance Statement

Nuclear‐encoded pentatricopeptide repeat (PPR) proteins perform site‐specific cytidine‐to‐uridine RNA editing in plant organellar transcripts. Their RNA‐binding specificity can be engineered, enabling targeted *de novo* RNA editing. However, it remains challenging to predict target specificity accurately, which is why empirical testing methods are required. Here, we developed a rapid and efficient *in vivo* assay to assess the RNA target selectivity of native and engineered PPR editing factor variants. Transient expression of the PPR editor in the cytosol of *Nicotiana benthamiana* followed by RNASeq enables empirical analysis of RNA target sequences. This figure was partly created with BioRender.

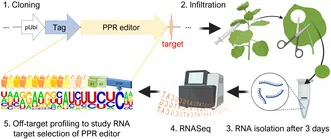

## BACKGROUND

Pyrimidine RNA editing is a hallmark of gene expression in the endosymbiotic organelles of land plants. Whereas uridine‐to‐cytidine (U‐to‐C) editing is restricted to hornworts, lycophytes, and ferns, cytidine‐to‐uridine (C‐to‐U) editing is almost ubiquitous (Freyer et al., 1997; Guo et al., 2015; Knoop, 2023; Steinhauser et al., 1999).

Nuclear‐encoded pentatricopeptide repeat (PPR) proteins of the PLS subfamily are at the core of the organellar C‐to‐U RNA editing machinery (Barkan & Small, 2014; Kotera et al., 2005). Serving as specificity determinants, they identify and bind their target RNA/s through their PPR arrays, positioning intrinsic or *in trans*‐acting DYW‐type cytidine deaminase domains (Andrés‐Colás et al., 2017; Oldenkott et al., 2019; Salone et al., 2007).

The PPR arrays of editing factors typically consist of 35 amino acid long P‐type, 35–36 amino acid long L‐type, and 31 amino acid long S‐type PPRs, arranged in P‐L‐S triplets (Cheng et al., 2016). C‐terminally, they culminate in a distinct PLS triplet (P_2_L_2_S_2_), followed by two 34 amino acid long ‘extension’ repeats E1 and E2, and eventually a truncated or complete DYW domain. A notable exception of the classic editing factor arrangement was found, for example, in the lycophyte *Selaginella moellendorffii*, where arrays frequently feature consecutive S‐type PPRs, as well (Cheng et al., 2016).

Specificity is obtained through a 1:1 interaction of PPRs and nucleotides (Yin et al., 2013), following a well‐described code (Barkan et al., 2012; Shen et al., 2016; Takenaka et al., 2013; Yagi et al., 2013; Yan et al., 2019). In particular, the fifth and last amino acids of P‐ and S‐type PPRs define to which nucleotide they bind. Their modular and programmable mode of interaction makes PPR proteins exciting candidates to engineer biotechnological tools.

However, compelling data demonstrates that editor‐RNA interactions are more complex than initially anticipated. For example, (i) the importance of PPR‐RNA binding, and therefore mismatch tolerance, is governed by the position of a given PPR within the array (Kindgren et al., 2015; Miranda et al., 2018), (ii) the overall fit between a PPR array and its nucleotide target sequence/s does not necessarily correlate with RNA editing rates (e. g., in case of moss editing factor PpPPR56; Ohtani et al., 2010), and (iii) L‐type PPRs, E repeats and DYW domains may also be involved in RNA target selectivity (Bayer‐Császár et al., 2023; Ichinose & Sugita, 2018; Okuda et al., 2014; Ruwe et al., 2019; Yan et al., 2017).

Recently, different designs of PPR arrays were engineered and applied for targeted RNA base editing *in planta* (Kwok van der Giezen et al., 2024; Mathieu et al., 2025; Royan et al., 2021). Despite the success, these pilot studies revealed that the editing efficiency and specificity of synthetic editors need to be improved. A more comprehensive understanding of the editing mechanism is vital for improving artificial PPR editing factors in the future.

To facilitate the testing of PPR editing factor performance and specificity, different heterologous assays in *E. coli* (Oldenkott et al., 2019), human cell cytosol (Lesch et al., 2022), plant cytosol (Thielen et al., 2024), bacterial cell‐free lysate (Dennis et al., 2025), and yeast cytosol (Ramanathan et al., 2025) have been developed. A key advantage of eukaryotic cytosolic assays is the availability of large and diverse nuclear transcriptomes that offer a plethora of putative off‐target editing targets at high redundancy. Profiling of such off‐targets enables empirically defining editing factor specificities with a high degree of accuracy. As of now, the most extensive datasets have been obtained from the inducible expression of editing factors of the model moss *Physcomitrium patens* in its cytosol (Thielen et al., 2024). However, this assay relies on the generation of stable transgenic lines. As such, it is time‐, labor‐, and cost‐intensive, precluding fast, flexible, and high‐throughput experimentation. To address those shortcomings, we developed an improved rapid plant cytosolic assay using transient expression in *Nicotiana benthamiana* leaves, which minimizes the required experimental efforts from several months to a few days. We analyzed the cytosolic editing activities of the well‐studied *Physcomitrium* editing factor PpPPR56 to validate and benchmark the setup. The specificity profile of PpPPR56, inferred from more than 1500 off‐target editing sites detected in the Nicotiana cytosol, closely matched previous results (Thielen et al., 2024), underscoring the reliability of the transient approach. Allowing for the rapid testing of native and artificial editing factor variants, the assay removes a critical methodological bottleneck for our understanding of the mechanisms underlying organellar RNA editing and the rational design of artificial RNA editing strategies.

## RESULTS

### Transient expression of eYFP‐PpPPR56 in the cytosol of *Nicotiana benthamiana*

PpPPR56 is one of nine organellar RNA editing factors of the model moss *Physcomitrium patens*. It comprises 14 PLS‐type PPRs and a carboxyterminal DYW‐type cytidine deaminase domain and performs site‐specific editing of two cytidines in the *nad3* (nad3eU230SL) and *nad4* (nad4eU272SL) transcripts in the mosses' mitochondria (Ohtani et al., 2010; Figure 3, top panel). We recently showed that PpPPR56 is also functional in the cytosol of *Physcomitrium*, but only upon a short burst of overexpression as achieved by an inducible expression system (Thielen et al., 2024). Apart from a co‐transcribed *nad4* target variant, PpPPR56 edited more than 900 additional cytidines in the *Physcomitrium* cytosolic transcriptome. Profiling of these off‐targets helped us to map the editing factor's RNA‐binding landscape (Thielen et al., 2024). Here, we set out to optimize a transient expression assay using the model angiosperm *Nicotiana benthamiana* with the aim to deliver comparable depth of insight yet at a fraction of the experimental effort and time.

Following the rationale of previous successful heterologous expression experiments (Lesch et al., 2022; Oldenkott et al., 2019; Thielen et al., 2024), we chose to express a truncated version of PpPPR56, PPR56:n14L14‐DYW (including 14 amino acids upstream (‘n’) of the N‐terminal L‐14 PPR and extending to the end of its DYW domain) that lacks the mitochondrial presequence. Instead, the coding sequence (CDS) of the enhanced yellow fluorescent protein (eYFP) was added upstream to enable tracking the expression of the recombinant protein within the leaf tissue and over time. The CDSs were cloned into the pSS02 vector downstream of the *Arabidopsis thaliana* *UBIQUITIN10* promoter.

Constructs were infiltrated into leaves of *Nicotiana benthamiana* using *Agrobacterium tumefaciens* GV3101 cells diluted to an OD_600_ of 0.3, and leaf discs were excised daily up to 5 days post‐infiltration (dpi). Cytosolic expression of eYFP‐PpPPR56 constructs was verified by confocal microscopy (Figure 1A). YFP signals peaked at 3 dpi, and no considerable increases were observed thereafter (Figure 1B). Even 5‐day post‐infiltration, leaves expressing eYFP‐PpPPR56 did not show any signs of necrosis (Figure S3).

**Figure 1:**
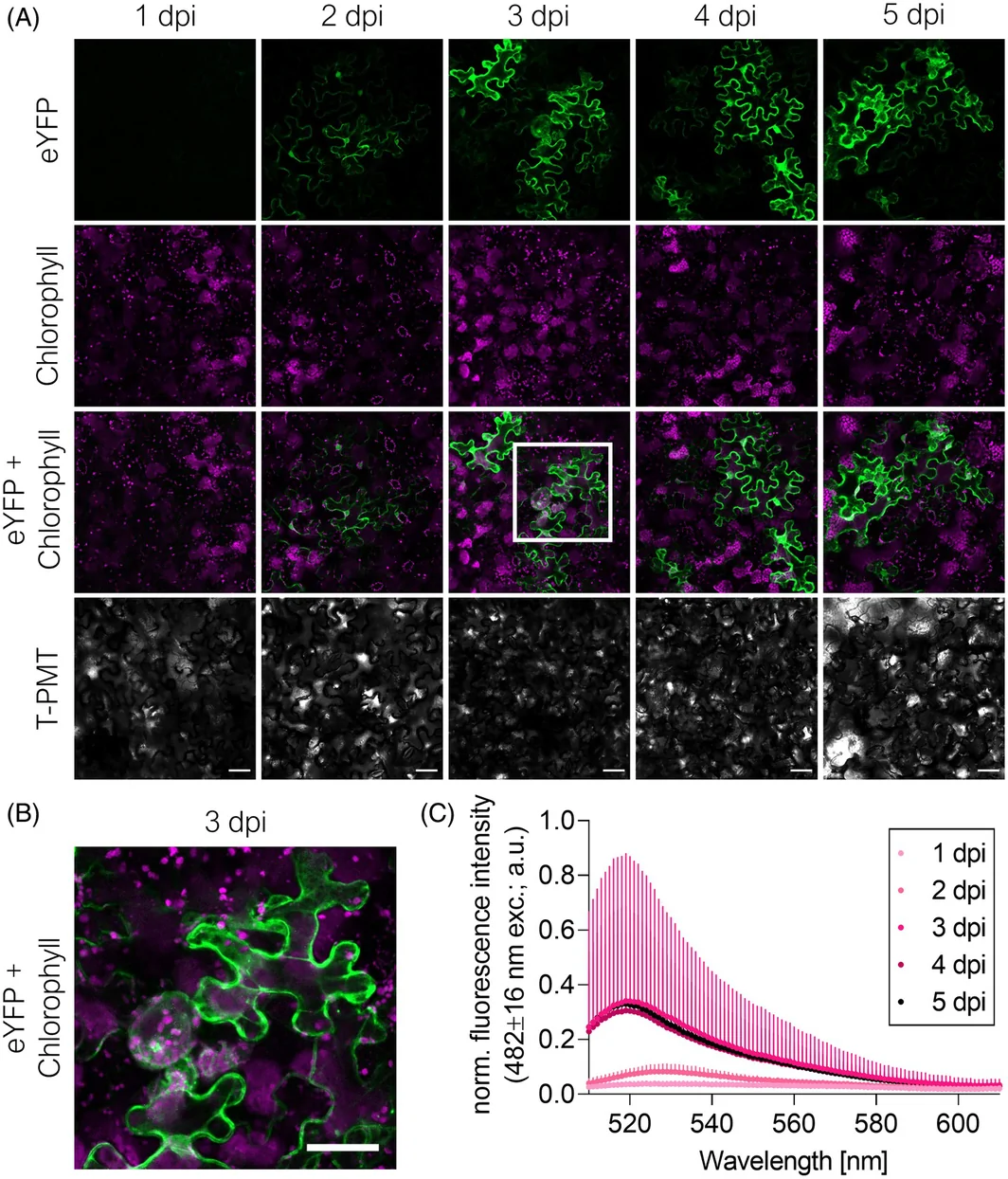
Expression of eYFP‐PpPPR56 in the *Nicotiana benthamiana* cytosol. (A) Confocal microscopy images of eYFP‐PpPPR56 expressed in the nucleo‐cytosol of transiently infiltrated *Nicotiana benthamiana* leaves at 1 to 5 days post‐infiltration (dpi). T‐PMT (transmission photomultiplier tube) resembles brightfield image. Magnification, controls and replicates are shown in Figure S1. Excitation wavelength: 514 nm (eYFP). Emission detection wavelengths: 517–570 nm (eYFP), 651–715 nm (chlorophyll). Scale bar = 50 μm. (B) Magnified view of the area marked in (A) (white box). Scale bar = 50 μm. (C) Normalized fluorescence spectra of eYFP‐PpPPR56 at 1 to 5 days post‐infiltration (dpi). Excitation wavelength: 482 ± 16 nm. Emission detection wavelengths: 510–610 nm. Fluorescence (in arbitrary units, a.u.) was normalized to chlorophyll autofluorescence at 610 nm. Controls expressing a mutant version of eYFP‐PPR56 and p19, as well as p19 only, are shown in Figure S2. *N* = 4.

### C‐to‐U RNA editing by PpPPR56 in the cytosol of *Nicotiana benthamiana*

After demonstrating that eYFP‐PpPPR56 can transiently accumulate in the cytosol of *N. benthamiana*, we aimed at testing whether the recombinant protein is capable of RNA editing. A variant of PpPPR56's native *nad4* target encoding 40 nucleotides up‐ and 5 downstream of the editing site encoded in the 3′‐UTR of the respective protein coding sequence was used for straightforward readout (see Figure 2A). eYFP‐PpPPR56:n14L14‐DYWmut with a mutation in the zinc binding motif that inhibits cytidine deamination (Lesch et al., 2022) was included as a negative control. A third construct encoding PpPPR56:n14L14‐DYW with an N‐terminal combined HA (hemagglutinin)‐His_6_‐maltose binding protein (MBP) tag (‘HHM’) was included (Figure 2A), as it drove most efficient editing of the cytosolic *nad4* target in the human cell cytosol (Lesch et al., 2022).

**Figure 2:**
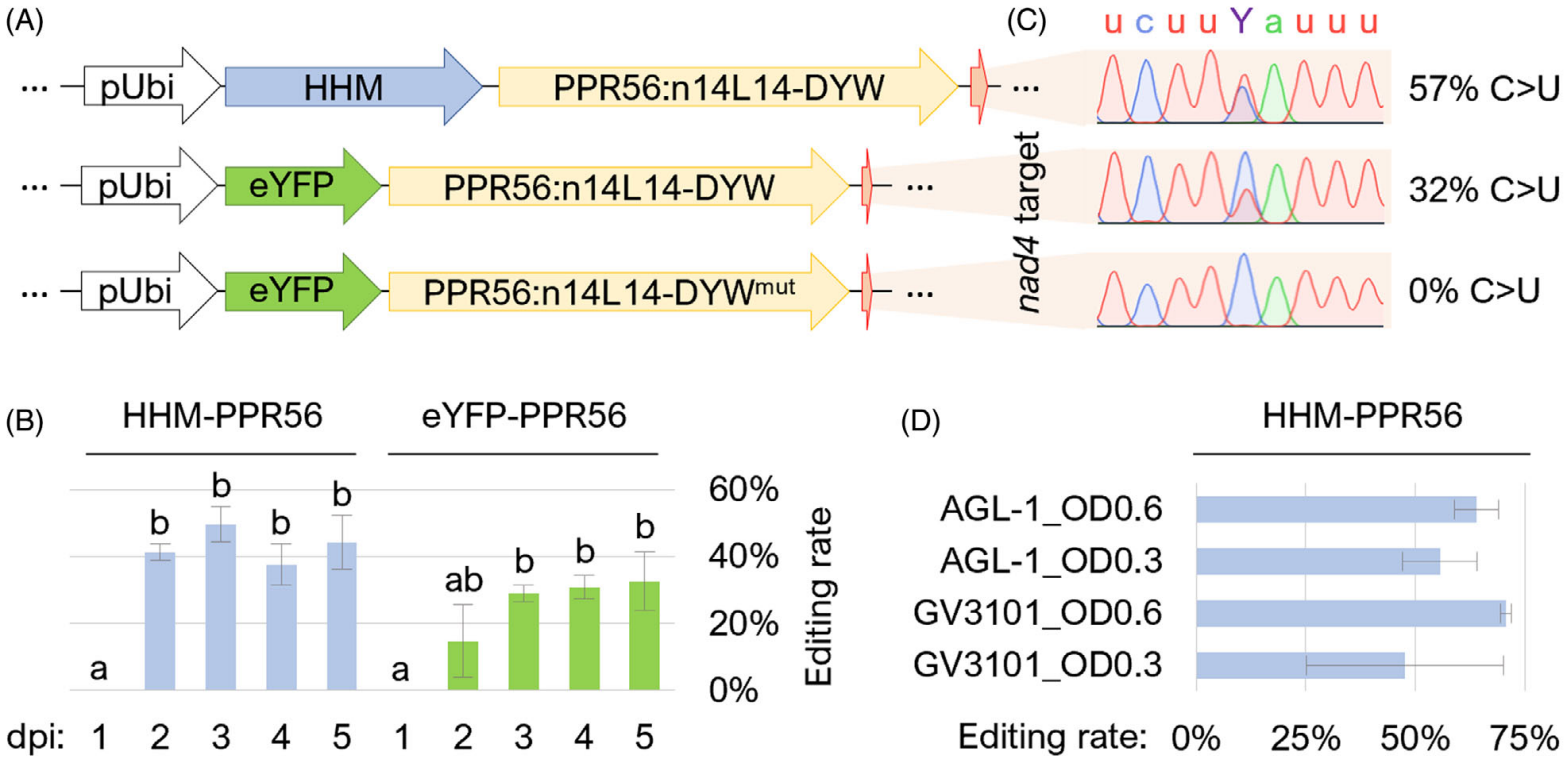
PpPPR56 is functional in the *Nicotiana benthamiana* cytosol. (A) The N‐terminally truncated coding sequence (CDS) of editing factor PpPPR56, retaining 14 amino acids upstream of the first PPR repeat L‐14, and its target sequence (red arrow; 40 base pairs up‐ and 5 downstream nad4eU272SL) were cloned into pSS02 downstream of the ubiquitin promoter (pUbi) and fused to either HA‐His_6_‐MBP (HHM) or enhanced yellow fluorescent protein (eYFP) CDSs, respectively. A mutant version eYFP‐PpPPR56^mut^ featuring a cysteine–alanine mutation in the conserved CxD **C** H zinc binding motif of its cytidine deaminase domain, which was shown to abolish editing activities (Lesch et al., 2022) was included as a control. (B) Constructs were infiltrated into 4‐ to 5‐week‐old *Nicotiana benthamiana* leaves using *Agrobacterium tumefaciens* strain GV3101 diluted to an OD_600_ of 0.3. Leaves were harvested 1 to 5 days post‐infiltration (dpi) and RNA editing rates at the co‐transcribed *nad4* target were determined. Mean values and standard deviations for three replicates (leaves) are shown, and all replicates are listed in Table S1. Statistical significance was assessed using one‐way anova followed by Tukey *post hoc* test in OriginPro® 2025 (*P* < 0.05). (C) Exemplary chromatograms (SnapGene Viewer, https://www.snapgene.com) displaying C‐to‐U (C>U) RNA editing at co‐transcribed targets, at 3 dpi. (D) HHM‐PpPPR56 was infiltrated into 4‐ to 5‐week‐old *Nicotiana benthamiana* leaves using *Agrobacterium tumefaciens* strain GV3101 or AGL‐1, diluted to an OD_600_ of 0.3 or 0.6, respectively, and editing rates at the co‐transcribed *nad4* target were determined. Mean values and standard deviations for three replicates (leaves) are shown, and all replicates are listed in Table S1.

Indeed, partial editing at co‐transcribed *nad4* targets could be verified as early as two dpi in leaves expressing HHM‐PpPPR56:n14L14‐DYW and eYFP‐PpPPR56:n14L14‐DYW, whereas no editing was detected under eYFP‐PpPPR56:n14L14‐DYW^mut^ expression in any sample (Figure 2B,C; Table S1), even though expression of the latter was verified by confocal microscopy and a plate reader‐based fluorescence analysis (Figures S1C,D and S2A). Independent of the incubation time after transfection, HHM‐PpPPR56:n14L14‐DYW outperformed eYFP‐PpPPR56:n14L14‐DYW in terms of editing capacity. Editing rates did not significantly increase after two dpi, and rates were highest with HHM‐PpPPR56:n14L14‐DYW at three dpi (Figure 2B).

To further optimize the cytosolic RNA editing rates of PpPPR56 in *N. benthamiana*, alternative strains and dilutions of *Agrobacterium tumefaciens* were used for infiltration of HHM‐PPR56:n14L14‐DYW. A switch to the AGL‐1 strain did not cause a notable improvement of editing rates at 3 dpi (Figure 2D). In contrast, editing was slightly increased in leaves infiltrated with cells diluted to an OD_600_ of 0.6 instead of 0.3.

### More than 1500 off‐targets of PpPPR56 in the *N. benthamiana* cytosolic transcriptome

Previous studies demonstrated that PpPPR56 activities were not restricted to co‐transcribed native target sequences in the eukaryotic cytosol (Lesch et al., 2022; Thielen et al., 2024). To investigate off‐target editing activities of PpPPR56 in the cytosol of *N. benthamiana*, its endogenous transcriptome was screened for single nucleotide variants (SNV). For that purpose, RNA was isolated from leaves expressing p19 only (one replicate), p19 and eYFP‐PPR56:n14L14‐DYW^mut^ (one replicate), and p19 and eYFP‐PpPPR56:n14L14‐DYW or HHM‐PpPPR56:n14L14‐DYW (three replicates, see Table S2 for details) and sent for RNA sequencing.

Co‐transcribed *nad4* targets were edited in 73, 45, and 22% of reads in replicates one (HHM‐PpPPR56:n14L14‐DYW, OD_600_ = 0.6), two (HHM‐PpPPR56:n14L14‐DYW, OD_600_ = 0.3), and three (eYFP‐ PpPPR56:n14L14‐DYW, OD_600_ = 0.3), respectively, but not in the eYFP‐PPR56:n14L14‐DYW^mut^ control, confirming the values determined by RT‐PCR and Sanger sequencing (Table S2). Notably, more than 1500 off‐target C‐to‐U RNA editing events were verified in the leaf nucleo‐cytosolic transcriptome after 3 days of PPR56 construct expression (Figure 3). In some cases, off‐target editing rates even exceed *nad4* ‘on‐target’ editing (Figure 3B).

**Figure 3:**
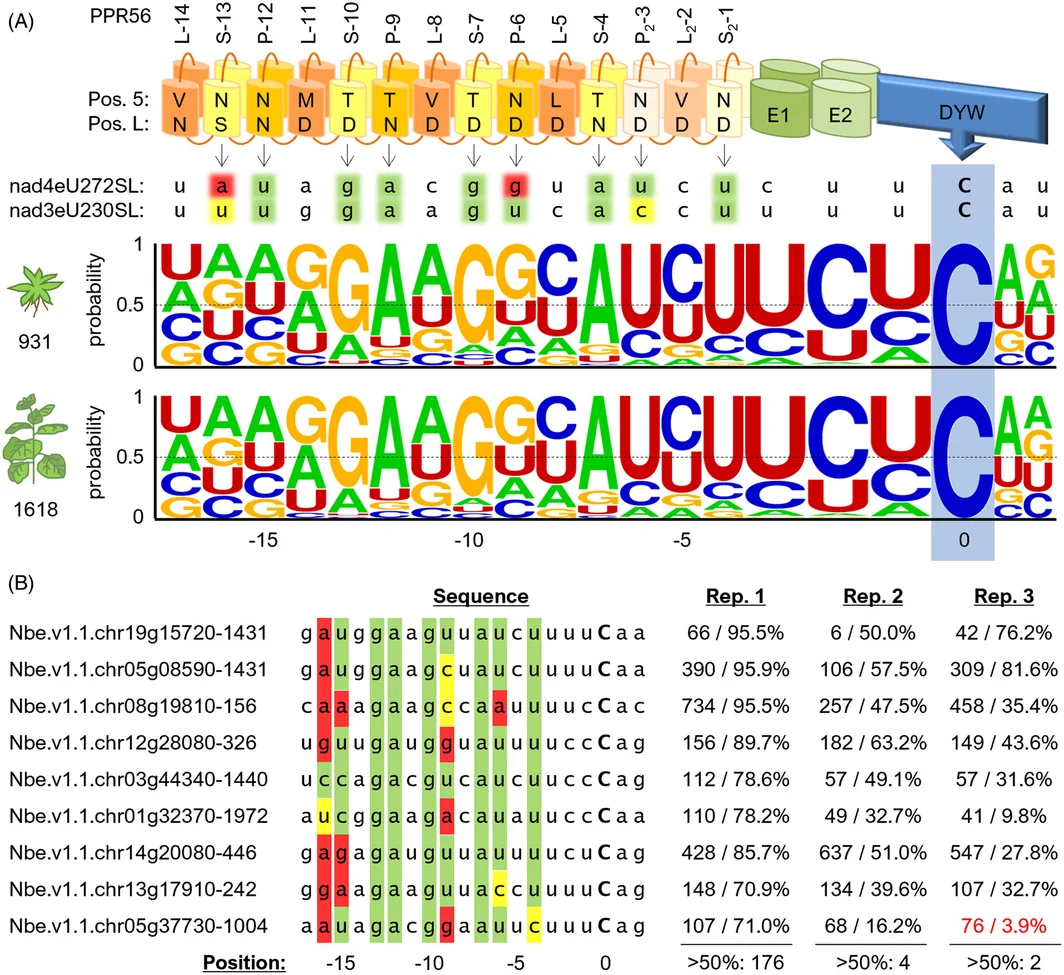
Transient cytosolic expression of PpPPR56 in *Nicotiana benthamiana* causes sequence‐specific C‐to‐U RNA editing at more than 1500 sites within its endogenous cytosolic transcriptome. (A) Sequence environments including 17 nucleotides up‐ and two downstream of these off‐target (OT) editing sites (position 0, blue shading) are shown as sequence logos (Crooks et al., 2004). The PPR array of PpPPR56 displaying the specificity‐determining fifth (Pos. 5) and last (Pos. L) amino acids is aligned to the region upstream of the editing position, with PPR S_2_‐1 juxtaposed to position ‐4. Native target sequences of PpPPR56 in the mitochondrial *nad3* and *nad4* transcripts of *Physcomitrium patens* are shaded following the PPR‐RNA‐binding code (Barkan et al., 2012). A sequence logo summarizing 931 off‐targets of PpPPR56 in the cytosolic transcriptome of the moss *Physcomitrium patens* is shown for comparison (Thielen et al., 2024). (B) Sites exhibiting >50% editing in reads summoned for replicates in which they were called. Upstream nucleotides are shaded as above. Coverage and editing is given for each individual replicate (Rep.). Nbe.v1.1.chr05g37730‐1004 was not called in replicate three (red font). 176, four and two sites are edited in >50% of reads in replicates one, two, and three, respectively. Detailed information about all off‐targets is shown in Table S3.

A sequence logo (Crooks et al., 2004) displaying the off‐target sequence environments reveals striking similarity to the current benchmark dataset obtained from 931 nucleo‐cytosolic off‐targets of PpPPR56 in *P. patens* (Thielen et al., 2024). To sum up, (i) nucleotide specificities of most S‐ and P‐type PPRs match the PPR‐RNA‐binding code (Barkan et al., 2012), with the exceptions of PPRs P‐6ND, P‐12NN and S‐13NS, (ii) positions opposite of PPRs L_2_‐2VD and L‐5LD exhibit notable pyrimidine predominance, and (iii) nucleotides ‐1, ‐2, and ‐3 directly upstream of the editing cytidine are almost exclusively pyrimidines—a selectivity likely not elicited by the PPR array, but rather by the C‐terminus of PpPPR56 (Figure 3A).

Interestingly, over three times as many off‐targets were detected in both HHM‐PpPPR56:n14L14‐DYW replicates, than in the eYFP‐PpPPR56:n14L14‐DYW replicate (Table S2). Furthermore, leaf infiltration with Agrobacterium cells diluted to an OD_600_ of 0.6 instead of 0.3 correlates with (i) higher HHM‐PpPPR56:n14L14‐DYW expression levels (5.4 versus 1.1% plasmid mapping reads), (ii) more off‐targets with editing rates above 50% (176 versus 4), and (iii) higher mean off‐target editing rates (24.3 versus 6.8%; compare Tables S2 and S3; Figure 3B). However, conclusions based on individual replicates should be drawn carefully, especially because of the transient nature of the assay.

## DISCUSSION

Following the discovery of the first chloroplast RNA editing factor in 2005 (Kotera et al., 2005), the identification of an increasing number of PPR editors and their organellar target sites via reverse genetics (see, e.g., EdiFacts database: http://www.prepact.de/prepact‐main.php; Lenz et al., 2018), combined with *in vitro* PPR‐RNA‐binding experiments, soon enabled the decryption of the specificity‐determining PPR‐RNA‐binding code (Barkan et al., 2012; Takenaka et al., 2013; Yagi et al., 2013; Yan et al., 2019). Nevertheless, there has been increasing evidence that editor‐RNA interactions are more complex than initially anticipated. Understanding the determinants of the target selection mechanism of PPR editors remains a central challenge, particularly if we are to develop specific and efficient biotechnological tools for the flexible manipulation of organellar gene expression at the RNA level (Kwok van der Giezen et al., 2024).

The emergence of setups for the co‐expression of editing factors and RNA targets in *E. coli* (Oldenkott et al., 2019), *in vitro* (Dennis et al., 2025; Hayes & Santibanez, 2020), and in the cytosol of cultured human cells (Ichinose et al., 2022; Lesch et al., 2022) and baker's yeast (Ramanathan et al., 2025) has been instrumental in accelerating our understanding of PPR editing factor function. Notably, the eukaryotic cytosols offer larger and more diverse transcriptomes than prokaryotic cytosols or eukaryotic organelles, resulting in substantial off‐target editing (see Table 1). Clearly, such datasets of editable sequences provide rich insight into the RNA target selection of the editors expressed. The largest off‐target datasets by far to date were obtained when expression of DYW‐type editors PpPPR56, PpPPR65, and PpPPR78 of the model moss *Physcomitrium patens* was induced in its cytosol for 24 h (Table 1; Thielen et al., 2024). Large datasets such as these represent a powerful resource to dissect the determinants underlying editing specificity and efficiency and to refine predictive models for synthetic editing factors. However, their generation via the establishment of stable transgenic *Physcomitrium* lines remains experimentally demanding and time‐consuming (see Figure 4, top panel). This critical bottleneck has called for an optimized assay enabling a faster generation of comparable datasets.

**Table 1:** Off‐targets of *Physcomitrium patens* editing factors PpPPR56, PpPPR65 and PpPPR78 in different heterologous expression systems

Genetic background		Number of sites edited		
		PpPPR56	PpPPR65	PpPPR78
*P. patens* mitochondrion (native)		2	1	2
Off‐targets	*E. coli*	79 Oldenkott et al. (2019)	7 Oldenkott et al. (2019)	4 Lesch et al. (2024)
	IMR‐90 human cell cytosol	906 Lesch et al. (2022)	(7)^a^ Lesch et al. (2022)	n/a
	*S. cerevisiae* cytosol	125 Ramanathan et al. (2025)	n/a	5 Ramanathan et al. (2025)
	*P. patens* cytosol	931 Thielen et al. (2024)	64 Thielen et al. (2024)	108 Thielen et al. (2024)
	*N. benthamiana* cytosol	1618 This study	n/a	n/a

Native RNA editing sites of *Physcomitrium patens'* editing factors PpPPR56, PpPPR65, and PpPPR78 (Ohtani et al., 2010; Rüdinger et al., 2011, 2009), and off‐targets identified through various heterologous expression setups (Lesch et al., 2024, 2022; Oldenkott et al., 2019; Ramanathan et al., 2025; Thielen et al., 2024) are shown. As off‐target detection pipelines differed slightly between the studies, direct comparisons should be drawn with consideration to those differences.

^a^ Result not immediately comparable to PpPPR56 (Lesch et al., 2022).

**Figure 4:**
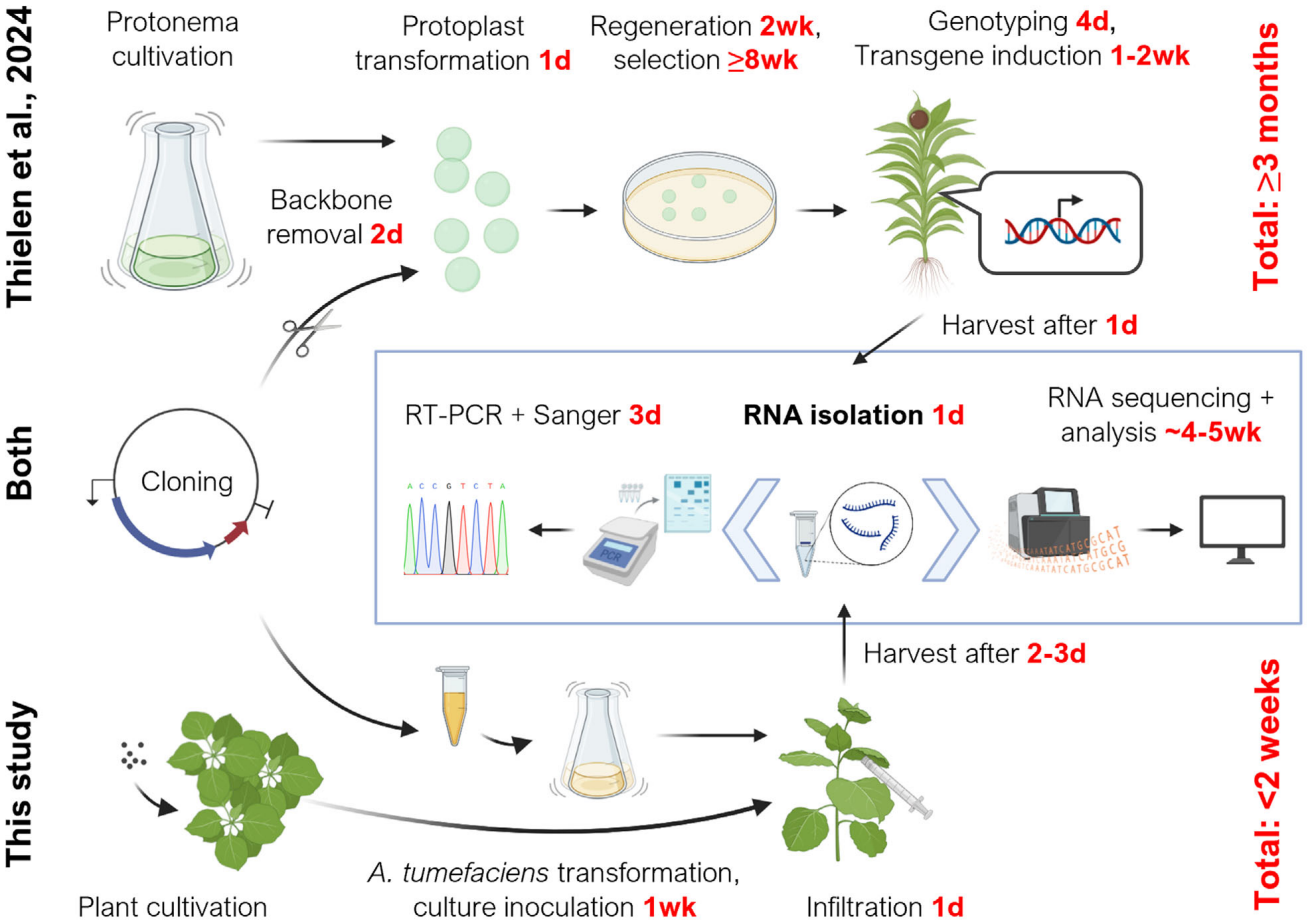
A time‐optimized assay to examine RNA target selection of PPR editing factors in *Nicotiana benthamiana*. This schematic workflow compares two *in planta* assays for the assessment of PPR RNA editing factor specificity. Estimates for the duration of individual steps are noted in red (d = day, wk = week). In contrast to a previously published assay, which is based on the generation of transgenic *Physcomitrium patens* lines and the inducible expression of cytosolic editing factor constructs (Thielen et al., 2024; top panel), a more rapid assay was developed in this study (bottom panel). Experimentation is substantially accelerated by using transient expression in *Nicotiana benthamiana* leaves, rendering the generation, selection, and validation of stable transgenic lines obsolete. Ultimately, the time span from construct cloning to sample harvest for RNA preparation could thereby be reduced from at least 3 months to less than 2 weeks. The isolated RNA can subsequently be used to assess editing at co‐transcribed, known target sequences (blue box, left side) or be submitted for RNA sequencing to assess off‐target editing and RNA target specificity (blue box, right side). This figure was partly created with BioRender (https://BioRender.com/i4ho4bu).

Here, we present a rapid plant cytosolic setup based on transient expression in *Nicotiana benthamiana*. Our assay allows editing specificities to be assessed within days rather than months (see Figure 4, bottom panel). Using the well‐characterized editor PpPPR56, we demonstrate that the system faithfully reproduces previously reported editing specificities (Lesch et al., 2022; Oldenkott et al., 2019; Thielen et al., 2024) within a fraction of the time. Although not investigated in this study, we expect that other functionally autonomous editors, such as PpPPR65 and PpPPR78, which are active in multiple heterologous assay systems (Table 1; Lesch et al., 2022, 2024; Oldenkott et al., 2019; Ramanathan et al., 2025; Thielen et al., 2024), also function in the cytosol of *N. benthamiana*. An exciting future perspective would be to test whether our assay can be optimized to enable the reconstitution of more complex editosomes, such as those found in angiosperms (Sun et al., 2016).

However, there are also some limitations to the presented assay. Firstly, the detection of off‐target edits in native mRNAs of *N. benthamiana* and their apparent editing rates depend on the transformation efficiency and editing factor expression levels, which can vary between batches, leaves, or leaf areas (see Table S1; Bashandy et al., 2015; Tang et al., 2026). Therefore, comparison of off‐target amounts and editing rates between different constructs should be avoided when using transient expression. To account for transformation efficiency and expression levels, a fluorescence‐based normalization approach may be developed in the future. Also, the requirement of individual leaves as replicates still conflicts with real high‐throughput construct testing. Scaling down the assay to a well plate using protoplasts (Khan et al., 2026) may be an option for scaling the analysis toward high‐throughput in the future. However, protoplast transformation is technically more challenging and efficiencies can be low (Neubauer et al., 2022), variable, and highly dependent on various parameters (Shen et al., 2014). Cell sorting or even larger amounts of sequencing data may be required to mitigate low transformation efficiencies and allow the detection of lowly edited off‐targets.

To conclude, the assay introduced here removes a critical bottleneck for RNA editing research. For example, it may aid shedding light onto the unresolved question why PPR:nucleotide interaction is not exclusively pairwise (Dennis et al., 2025; Lesch et al., 2022) and how neighboring PPRs and/or nucleotides affect editing. It may further be exploited to map the determinants of the editability of cytidines in given sequence contexts, which are likely inherent of editing factor's C‐terminal domains (Maeda et al., 2022; Thielen et al., 2024; Yang et al., 2023), and help to understand, why PPRs in editing factors come in three ‘flavors’ (Knoop, 2020). Ultimately, such a level of mechanistic understanding will be needed for the rational design of synthetic factors for efficient and specific RNA editing.

## MATERIALS AND METHODS

### Cloning recombinant constructs to investigate cytosolic RNA editing

The coding sequences (CDSs) of HA‐His_6_‐MBP (HHM)‐ and eYFP‐PpPPR56 fusion proteins with downstream target sequences were amplified from plasmid clones (Lesch et al., 2022) using the Phusion proofreading polymerase (New England Biolabs GmbH, Frankfurt, Germany). Framing amplification primers (Table S4) featured overhangs for Gibson Assembly. PCR products were purified (Macherey Nagel, Düren, Germany) and inserted into the pMDC32 (Curtis & Grossniklaus, 2003)‐derivative pSS02 (Steinbeck et al., 2020) using the NEBuilder HiFi DNA Assembly kit (New England Biolabs GmbH). Constructs were verified through Sanger sequencing (Microsynth, Balgach, Switzerland).

### Plant material and cultivation

*Nicotiana benthamiana* plants were cultivated in the greenhouse at 22°C with a 16‐h/8‐h day/night cycle and approximately 400 μmol/m^2^/sec light intensity.

### Transient construct expression in *Nicotiana benthamiana*

Expression vectors were introduced into *Agrobacterium tumefaciens* GV3101 or AGL‐1 by cold shock. Plasmid presence was verified in individual bacterial clones through colony PCR (Taq mix, 7Bioscience, Neuenburg, Germany). About 5 ml pre‐cultures (LB + rifampicine + gentamycine + kanamycine) were grown overnight and used to inoculate main cultures. After 1 day of incubation at 28°C and 140 rpm, Agrobacteria were collected by centrifugation, resuspended in infiltration buffer (10 mm MES pH 5.6, 10 mm MgCl_2_, 150 μg/ml acetosyringone and 0.1% DMSO) and incubated for at least 2 h shaking at room temperature. Prior to infiltration, bacterial suspensions were diluted to an OD_600_ of 0.3 or 0.6.  4‐ to 5‐week‐old *N. benthamiana* plants were infiltrated on the abaxial side of the third and fourth youngest fully expanded leaves using a needleless syringe. Afterwards, plants were grown in the greenhouse for 1 to 5 days.

To study and quantify YFP expression, leaves were additionally infiltrated with pH2GW7 encoding cpYFP (Schwarzländer et al., 2011) as a control.

### Confocal microscopy

To verify construct expression in *Nicotiana benthamiana*, leaf discs were excised at 1 to 5 dpi, as shown exemplarily in Figure S4A. Confocal imaging was performed using a Zeiss LSM980 microscope with either a 10× Plan‐Apochromat/0.45 M27 or a 40× C‐Apochromat water immersion objective (Carl Zeiss Microscopy GmbH, Jena, Germany). YFP was excited at 514 nm (3% laser power). Emission was detected at 517–570 nm for eYFP/cpYFP and 651–715 nm for chlorophyll. Pinhole size was set to 0.78 (10×) and 0.86 (40×) Airy Units. Images were processed using ZEN Blue 3.4 software (Carl Zeiss, Jena, Germany).

### Quantification of YFP fluorescence

YFP fluorescence spectra of *Nicotiana benthamiana* leaf discs were measured in NUNC 96‐well plates (Thermo Fisher Scientific, Dreieich, Germany) as described by Multhoff et al. (2024). Fluorophores were excited at 482 ± 16 nm using a CLARIOstar® plate reader (BMG LABTECH, Ortenberg, Germany) with 50 flashes per well in 180 μl assay medium (10 mm MES, pH 5.8, 5 mm KCl, 10 mm MgCl_2_, 10 mm CaCl_2_). Emission was recorded from 510 to 610 nm in 1 nm increments at a focal height of 7 mm. Raw fluorescence data were normalized to chlorophyll autofluorescence at 610 nm.

### RNA editing analysis

Up to ca. 100 mg of leaf material was harvested (Figure S4B) and homogenized using metal beads and a tissue lyser II (Qiagen, Hilden, Germany). Nucleic acids were isolated using the Spektrum Plant total RNA kit (Sigma‐Aldrich, Taufkirchen, Germany) and DNA was depleted using DNase I (Thermo Fisher Scientific). cDNA was synthesized using the RevertAid Reverse Transcriptase (Thermo Fisher Scientific) and oligo‐d(T) primers. The cytosolic co‐transcribed targets were amplified using a Taq mix (7Bioscience, Neuenburg, Germany/ Bio‐Budget Technologies, Krefeld, Germany). After gel‐purification (Gel purification kit, Macherey Nagel) and Sanger sequencing (Macrogen Europe, Amsterdam, Netherlands), editing efficiency was calculated by dividing the T peak by the sum of T and C peak in the editing position using BioEdit 7.2.5 (Hall, 1999).

Replicates correspond to individual leaves. Statistical significance was assessed using one‐way anova followed by Tukey *post hoc* test in OriginPro® 2025 (OriginLab Corporation, Northampton, MA, USA; *P* < 0.05).

### Total transcriptome sequencing in *Nicotiana benthamiana*

*N. benthamiana* plants were infiltrated as above, and 100 mg leaf material harvested. Total RNA was isolated as above using the Spektrum Plant total RNA kit (Sigma) and subsequent DNaseI treatment (Thermo Fisher Scientific) was executed to eliminate potential DNA carryover. Library preparation with polyA‐RNA enrichment and Illumina sequencing (≥20 G raw data per sample, 150 bp paired‐end) were performed by Novogene (Cambridge, UK). A complete list of RNASeq samples is given in Table S2. Data were deposited in the NCBI SRA (National Center of Biotechnology Information Short Reads Archive) database under BioProject accession PRJNA1426925.

### Identification of nucleo‐cytosolic off‐targets in *Physcomitrium patens*

*Nicotiana benthamiana* RNASeq raw data quality was assessed using FastQC (https://www.bioinformatics.babraham.ac.uk/projects/fastqc/). Adaptors and low‐quality sequences were removed using Trimmomatic 0.39 with the options ILLUMINACLIP2:30:10, LEADING:15, TRAILING:15, SLIDINGWINDOW:4:24 and MINLEN:80 (Bolger et al., 2014). Reads mapping to the expression plasmid sequence were removed using BBDuk of the BBTools suite (Bushnell B., sourceforge.net/projects/bbmap) with default settings and the options *k* = 31 and hdist = 2. To determine editing rates at the co‐transcribed *nad4* target, these reads were mapped to the plasmid using BBMap (maxindel = 200 k, mdtag = true, sam = 1.4, subfilter = 3 and pairedonly = true). SAM files were converted into BAM format (samtools view), deduplicated (samtools fixmate, samtools markdup), and coordinate‐sorted (samtools sort; Samtools 1.10; Li et al., 2009). Base counts at the editing site were extracted from the samtools mpileup output.

To generate a custom reference, trimmed reads of a p19 control sample were mapped to *Nicotiana benthamiana* Nbe_v1.1_cds.fa (https://nbenthamiana.jp/downloads; Kurotani et al., 2025) using BBMap of the BBTools suite (Bushnell B., sourceforge.net/projects/bbmap/) with options maxindel = 200 k, mdtag = true, sam = 1.4 and pairedonly = true. The resulting SAM file was converted into BAM format using Samtools (v1.10; Li et al., 2009). An alignment‐based Salmon quantification with standard settings (Patro et al., 2017) was performed to estimate read abundance per transcript. A subset FASTA file containing only transcripts with an estimate of at least ten mapped read pairs (NumReads ≥10) was used as reference in all subsequent analyses.

The trimmed RNA reads of each sample were mapped to the custom reference using BBMap with the options maxindel = 200 k, mdtag = true, sam = 1.4, subfilter = 3 and pairedonly = true. The output SAM files were converted into BAM format (samtools view), deduplicated (samtools fixmate, samtools markdup), coordinate‐sorted (samtools sort), and indexed (samtools index) via Samtools 1.10 (Li et al., 2009).

SNPs between sample RNAs and cytidines in the reference were determined using the call‐1 option of JACUSA2 (Piechotta et al., 2022) with the options ‐m 3; ‐q 25; ‐T 1.56; ‐a I,M,B,Y and ‐f V. SNPs that were called in two or more replicates expressing PpPPR56, but not in the control samples (p19/PpPPR56 loss‐of‐function), were selected by a custom‐made R script (kindly provided by Simon Zumkeller). RNA editing at specific sites was defined as the ratio of edited to total RNA reads, averaged for the respective replicates, in which the SNPs were called. Only transcript‐level SNPs affecting cytidines with (i) a pyrimidine content of ≥99%, (ii) a C‐to‐U conversion rate of ≥1.5% and (iii) a cumulated coverage of ≥30, averaged for the respective replicates, in which the SNPs were called, and with (iv) a coverage of ≥20 and a uridine content of <0.5% in the p19 control, were considered further. Individual SNPs in questionable mapping regions were excluded after manual evaluation of read alignments using Tablet (Milne et al., 2013). Off‐targets were labeled and the sequences spanning 30 nucleotides up‐ and five downstream of the edited cytidines were extracted with a custom‐made R script (established with help of Simon Zumkeller) using R v3.13 (R Core Team, https://www.R‐project.org) and RStudio v2023.09.0 (RStudio Team, https://www.rstudio.com). A sequence logo was created using the web‐based WebLogo application (https://weblogo.threeplusone.com/; Crooks et al., 2004).

### Use of BioRender

The illustration tool BioRender (https://www.biorender.com/) was used to create the Graphical Abstract and Figure 4, and publication licenses have been obtained (https://BioRender.com/i4ho4bu, https://BioRender.com/0d9h21l).

## AUTHOR CONTRIBUTIONS

EL designed the research with input from all co‐authors; EL and DB performed the experiments and analyzed the data; TI and MS supervised the research; EL, MS, and TI acquired funding; and EL wrote the manuscript with contributions from all co‐authors.

## CONFLICT OF INTEREST

The authors declare no competing interests.

## Supporting information


**Figure S1.** Confocal microscopy images of eYFP‐PpPPR56 and controls in the *Nicotiana benthamiana* cytosol.
**Figure S2.** YFP fluorescence intensities in infiltrated *Nicotiana benthamiana* leaves.
**Figure S3.** Macroscopic leaf phenotype.
**Figure S4.** Sampling leaf material.

**Table S1.** List of individual editing replicates.

**Table S2.** Samples used for RNASeq studies.

**Table S3.** Detailed list of identified off‐targets of PpPPR56 in the cytosol of *Nicotiana benthamiana*.

**Table S4.** Oligonucleotides used in this study.

## Data Availability

RNA sequencing data were deposited in the NCBI SRA (National Center of Biotechnology Information Short Reads Archive) database under BioProject accession PRJNA1426925.
