# Supplementary material for: A rapid and efficient in planta assay for assessment of RNA editing factor performance
Source: Plant J. 2026 Oct 12;128(1):e71154. doi: 10.1111/tpj.71154 (PMC13656186; doi:10.1111/tpj.71154)
Supplement: Supplementary file 1 [file TPJ-128-0-s005.docx]

## Supplementary Figures

### Supplementary Figure S1: Confocal microscopy images of eYFP-PpPPR56 and controls in the *Nicotiana benthamiana* cytosol

Confocal microscopy images of p19+eYFP-PpPPR56 (A, B), p19+eYFP-PpPPR56mut (C, D), and p19+circularly permuted (cp) YFP (E, F) expressed in the cytosol of transiently infiltrated *Nicotiana benthamiana* leaves at one to five days post infiltration (dpi), and a control sample expressing p19, only (G, H). T-PMT (transmission photomultiplier tube) resembles brightfield image. Excitation wavelength: 514 nm (eYFP). Emission detection wavelengths: 517-570 nm (eYFP), 651-715 nm (chlorophyll). A, C, E, G: 10x Zoom, scale bar = 50 *µ*m. B, D, F, H: 40x Zoom, scale bar = 20 *µ*m.

### Supplementary Figure S2: YFP fluorescence intensities in infiltrated *Nicotiana benthamiana* leaves

Leaf discs were harvested at one to five days post infiltration (dpi). Mean fluorescence intensities (in arbitrary units, a.u.), normalized to the florescence emission at 650nm, of eYFP-PpPPR56mut (A) and circularly permuted (cp) YFP (B) expressed in the cytosol of transiently infiltrated *Nicotiana benthamiana* leaves, and a control sample expressing p19, only (C), are shown with standard deviations. A-C: N = 4, D: N=2.

### Supplementary Figure S3: Macroscopic leaf phenotype

At five dpi, leaves expressing editing factor constructs eYFP-PpPPR56 and eYFP-PpPPR56Mut (bottom panels) did not show enhanced welting or necrosis, compared to the controls expressing circularly permuted YFP or p19, only (top). Circular lesions were caused by the syringe during infiltration.

### Supplementary Figure S4: Sampling leaf material

Leaf sampling strategy for confocal microscopy and plate reader measurement (A), and for RNA isolation (B) is indicated exemplarily.
